# Abstract of an Artlcle on the “Anatomy and Physiology of the White Corpuscles of the Blood (Leucocytes)”

**Published:** 1859-05

**Authors:** C. Robin


### ABSTRACT OF AN ARTICLE ON THE "ANATOMY AND PHYSIOLOGY OF THE WHITE CORPUSCLES OF THE BLOOD (LEUCOCYTES)."

BY DR. C. ROBIN.

[Translated from the "Journal de la Physiologie," by E. L. HOLMES, M.D., of Chicago.]

The "white globules" (*Leucocytes*) is a name applied to those elements of the body, which are observed, either in the form of cells, or of free nuclei. These last, few in number, are spherical, without nucleoli, readily contracted and corrugated, by the action of acetic acid; the cells, of spherical form, are distinguished by irregularities of surface, and, above all, by being partially coagulated and dissolved by water, acetic acid, etc., which render them pale, and cause the appearance of nuclei, varying in number from one to four, when their granules have not been replaced by oil granules, which are often found in them.

§ I. *The distribution of the white globules in the system.*

These corpuscles comprise an element, which is found in the greatest number of animal tissues. Their anatomical and physiological history is of the greatest interest, on account of the variety of their forms, of the readiness with which they spring into existence and modify their structure. They are found in all parts of the body, where the red corpuscles are traced, and also in the lymph; in the smaller veins and arteries they usually lie in contact with their lining membranes. They are found in blood coagulated, either after death, or in a ligated artery, in an ordinary clot, obtained from bleeding, or from an apoplectic extravasation.

We obtain them from the splenic, renal, portal and pulmonary veins, perhaps in greater numbers, from these veins in the fœtus than in the adult; in the lymphatics of the neck, testis and groin, often in masses perceptible to the naked eye. It is in these various situations, that these bodies have been called respectively white globules of the blood, lymph, or chyle globules.

We meet them in all the fluids of the body, whether normal or abnormal.

They are especially numerous on the mucous surface of organs in a state of irritation, as, for instance, in the mucous of the bladder in certain conditions; they give the peculiar color to pus and so called "mucous;" tubercles also contain a perceptible portion of these globules.

### § *II. The form and composition of the white globules.*

The white globules assume forms varying with the places and conditions in which they are developed. They may be ovoid polyhedrous, or roughened like the surface of a mulberry. These forms soon, however, become perfectly spherical, when the causes which produced these differences of form have passed away. The various degrees of irritation and inflammation seem to exert great influence upon the forms of the white corpuscles; whenever these influences are alike in the different mucous membranes, the corpuscles all present precisely the same characteristics.

In a 1000 parts, there are found:

| | | | |
|---|---|---|---|
| Of water, | 790.00 | Fatty matter, | 26.50 |
| Salts, | 43.00 | Organic substances, | 140.00 |
| Iron, | a trace. | Albumen, | a trace. |
| Lactates, | undetermined. | Other substances | undetermined. |

### § *III. Origin of the white corpuscles.*

The white corpuscles are produced in all the various portions of the body in which they are found. Although inflammation is favorable to the production of the blastema, in which the corpuscles find their elements, it is not *necessary* to this development. Their presence in the vitreous humor, etc., prove that they may be developed in the tissues in which they are found, without the influence of glands. In several abnormal conditions, as intermittent fever, hypertrophy of the spleen and liver, typhoid fever, dysentery, and any form of cachexia, these corpuscles are developed in great numbers, producing the state known as leucocythemia.

### § *IV. Relation of the white to the red corpuscles.*

It was formerly believed that the white corpuscles are imperfectly developed red globules of the blood.

There is no evidence of this. Minute examinations of animals in an embryonic state, prove that the white corpuscles do not make their appearance till after the red globules are developed in considerable numbers.

### § *V. Development of the white corpuscles of pus.*

Like other elements of the system, the white corpuscles sometime appear in places where they do not normally exist: for instance, between the tissues of a muscle, in the form of abscess. Liquid tumors (abscess) are formed upon the same principle as solid tumors of different kinds, and, like these last, they invade the surrounding tissues, producing atrophy by pressure. Frequently, even bone is absorbed under the pressure of a tumor. The tissues surrounding an abscess are also gradually absorbed, till finally the pus is discharged, either through the integuments, or into one of the internal cavities. The development of the white corpuscles is generally far more rapid than cells which comprise solid tumors. That these pus globules are not lifeless red corpuscles, is shown by the fact, that they live, develope, become hypertrophied, and pass through all the modifications peculiar to a distinct class of organized cells.

We are able to watch the production of these corpuscles only on the surfaces deprived of the external integument.

In such conditions, we observe the following phenomena: at first there is an exudation of minute transparent drops, perhaps slightly colored with red corpuscles, forming a granular blastema; in the course of half an hour, sometimes less time, there are formed minute pale, transparent globules, granular, and often somewhat colored. The course of development goes rapidly forward till pus is formed, and when this has once taken place, a repetition of the same process continues.

### § *VI. Hypertrophy of the white corpuscles.*

The white corpuscles become hypertrophied whenever they are confined for a long time in collections, more or less minute. They become enlarged and granular in softened portions of the brain or spinal marrow; in epithelial and glandular tumors; in small purulent deposits in the lungs. In venous dilatations, sanguineous and synovial cysts, we find the white corpuscles granular and nearly double their usual size.

*VII. The destruction (disappearance) of the white globules.*

The duration of the white corpuscles is different in different regions of the body. Those of the lymph disappear in their passage through the blood. The *manner* in which they disappear is wholly unknown. That they become atrophied and rapidly pass away is seen in cases of leucocythemia, where the number of these corpuscles, largely increased, soon becomes normal.

There is no proof that they are changed to red corpuscles. In the very early stages of embryonic life, although the red corpuscles are very light colored, they do not resemble the white corpuscles.

It is well known that purulent collections sometimes become completely absorbed. The corpuscles often seem to become firmly molecular, and dissolved in the serum of the blood. But what, then, becomes of them at this stage of destruction is unknown.

